# Healthy Lifestyle Practices among Argentinian Vegetarians and Non-Vegetarians

**DOI:** 10.3390/nu11010154

**Published:** 2019-01-12

**Authors:** Rocio V. Gili, Sara Leeson, Evelyn M. Montes-Chañi, Daniel Xutuc, Ismael A. Contreras-Guillén, Gerardo N. Guerrero-Flores, Marcia C.T. Martins, Fabio J. Pacheco, Sandaly O.S. Pacheco

**Affiliations:** 1 Center for Health Sciences Research, School of Medicine and Health Sciences, Universidad Adventista del Plata, Libertador San Martín, 25 de Mayo 99, Entre Ríos 3103, Argentina; rociovictoriag@gmail.com (R.V.G.); saraleeson21@gmail.com (S.L.); evymar80@gmail.com (E.M.M.-C.); danielxutuc@gmail.com (D.X.); ismael.contreras@uap.edu.ar (I.A.C.-G.); gerardo.guerrero@uap.edu.ar (G.N.G.-F.); sandaly.oliveira@uap.edu.ar (S.O.S.P.); 2 Institute for Food Science and Nutrition, Universidad Adventista del Plata, Libertador San Martín, 25 de Mayo 99, Entre Ríos 3103, Argentina; 3 Department of Nutrition, Adventist University of São Paulo-UNASP/SP, Estrada de Itapecerica 5859, Jardim IAE, São Paulo 05858-001, Brazil; marciactm@yahoo.com.br

**Keywords:** vegan, vegetarian diets, lifestyle habits, dietary patterns, Argentina

## Abstract

Although current research has contributed to the promotion of whole-food plant-based diets, few studies have examined healthy vegan dietary and lifestyle factors, especially in South America. Therefore, we aimed at investigating the adherence to healthy vegan lifestyle habits among Argentinian vegetarians and omnivorous, using a recently developed vegetarian lifestyle index adapted to the vegan dietary pattern. Also, accessibility of vegetarian foods, and the proportion of household income spent on food were assessed in a cross-sectional approach with 1454 participants. The population was comprised of females (84.9%), singles (55.0%), young-adults (mean age 32.1, standard deviation (SD) = 13.6), employed (50.8%), with high educational levels (50.4%), and low prevalence of both tobacco smoking (7.0%) and frequent alcohol consumption (7.6%). The mean score of adherence to healthy vegan lifestyle habits was 6.64 (SD = 1.72), with higher scores indicating better adherence. Non-vegetarians (5.75; 95% confidence interval (CI), 5.61–5.89) had a significantly lower adjusted mean score compared to semi-(6.32; 95% CI, 6.17–6.47), pesco-(6.99; 95% CI, 6.59–7.39), lacto-ovo-vegetarians (7.10; 95% CI, 6.96–7.24), as well as vegans (8.59; 95% CI, 8.35–8.83). The mean proportion of household income spent on food was significantly lower among vegans compared with other dietary patterns. The whole population that was studied showed a low consumption of whole grains, legumes, vegetables, nuts, and seeds. Although vegans showed a better diet and lifestyle pattern there is a need to improve eating and lifestyle habits to address risk factors for non-communicable diseases in Argentina.

## 1. Introduction

There is a growing interest in deepening the understanding of the associations between lifestyle factors and health outcomes. Epidemiologic data have linked healthy dietary patterns and lifestyle habits with lower prevalence of several medical conditions and a quality aging [1,2,3,4,5,6,7,8]. Non-vegetarian dietary patterns, characterized by the frequent intake of animal foods have been associated with increased risk of developing several chronic diseases [9,10,11]. In contrast, vegetarian dietary patterns are generally associated with a more positive health status including lower total mortality [12], lower prevalence of weight gain or obesity [13,14,15], cardiovascular disease [16,17,18,19], metabolic syndrome [20,21], hypertension [22], diabetes [15,23,24], and some types of cancer [25,26]. More recent studies have suggested that a vegan dietary pattern may be associated with lower risk factors for some chronic diseases than other vegetarian patterns [27,28]. For instance, a vegan diet may offer additional protection against coronary heart disease [16,18], type 2 diabetes, and cancer [29,30,31].

The identification of specific dietary types of vegetarianisms has not been accurately defined in some epidemiologic studies. Vegetarian diets include a spectrum of dietary patterns which usually do not consume or consume limited amounts of animal meat, especially red meat, poultry and/or fish. Lacto-ovo-vegetarians are known to include dairy and eggs in their diet, and vegans exclude all kinds of animal meats or derived products. Some studies also include other categories of vegetarians such as semi- and pesco-vegetarians which include limited amounts of meat and/or fish in their diets [32]. More recent research suggests that the use of plant-based diets as a means of prevention and treatment of chronic diseases should be promoted through proper dietary recommendations and guidelines [27]. Thus, efforts are needed to evaluate vegan diets and to promote nutritional adequacy for vegetarians. This may be especially relevant in developing countries with high prevalence of non-communicable diseases (NCDs) [14,33,34,35] and few studies about vegetarian dietary patterns and lifestyles.

Along with proper nutrition, other lifestyle factors such as water intake, sunlight exposure, and daily exercise have been included in food guides for the promotion of comprehensive healthy lifestyles [36,37]. Although vegans tend to show healthier lifestyle habits than non-vegetarians [7,21,38,39], the avoidance of animal foods is not always accompanied by the adoption of other healthy dietary or lifestyle habits [40]. In fact, recent research shows that it is possible to have unhealthful plant-based diets with increased risk of coronary heart disease [41]. This may be particularly important when considering that research on vegan lifestyles is relatively recent and conducted mostly among highly-developed countries making it difficult to extrapolate some of the findings to other parts of the world. Usually, in developed contexts, people are exposed to better socioeconomic and educational experiences which may facilitate the adherence to vegan diets than people living in contexts of lower-and middle-income countries. In parallel, most cases of early death and disability on a global scale are associated with chronic diseases, but recent studies indicate that NCDs have a more negative impact on low-income and middle-income countries [42]. Argentina is among one of the South American countries with a high burden of morbidity and mortality associated with NCDs [34,35].

Previous findings from our studies showed a high prevalence of risk factors for NCDs in persons living in central areas of Argentina [43]. This includes unhealthy lifestyle habits associated with low consumption of fruits and vegetables, physical inactivity, high prevalence of overweight and obesity, tobacco smoking, and altered lipid and glucose blood serum profiles [43]. The impact of poor lifestyle habits on cardiovascular diseases and diabetes was shown in a 2018 study conducted in United Kingdom (UK) investigating genetic susceptibility in relation to lifestyle factors. Persons with high genetic risks for certain NCDs and poor lifestyle habits, compared with ideal lifestyles in the low genetic risk group, were several times at an increased risk of developing coronary artery disease (hazard ratio (HR) 4.45, 95% CI, 3.72–5.54), and diabetes (HR 15.46, 95% CI, 10.82–22.08) [44]. Another study with participants of the UK Biobank assessed the genetic risk of incident stroke, and the benefits of adhering to a healthy lifestyle including four factors: non-smoker, healthy diet, body mass index <30 kg/m^2^, and regular physical exercise. Unfavorable lifestyle, characterized by 1 or less healthy lifestyle habits, was associated with a 66% increased risk of stroke compared with a favorable lifestyle (3 or 4 healthy lifestyle habits) independently of genetic risk levels. Thus, adopting healthy lifestyle habits, may be the most affordable and effective strategy to address the health crisis associated with NCDs worldwide, especially in low-and middle-income countries [45]. Then, there exists an actual need to deepen the comprehension of vegetarian lifestyles in different contexts of the world, to identify risk and protective factors which support health promotion and prevention of NCDs.

The evaluation of individual’s lifestyle habits may be done using several approaches. More recently, researchers have designed new tools to access food intake in a composite mode [46]. This is the case of eating indexes. Considering the diversity of dietary patterns, a specific index was recently developed to assess vegetarian dietary patterns and lifestyle habits. The Vegetarian Lifestyle Index (VLI) [7] was developed based on the guidelines for healthful vegetarian diets [47] and was shown to accurately discriminate lifestyle habits between different dietary patterns [7]. This index may be applied in different settings, such as in nutritional research for evaluation of diet and lifestyle adherence in community-based interventions, population surveillance of lifestyle quality, and epidemiological research [7].

Therefore, this study aimed at investigating the adherence to healthy vegan lifestyle habits among Argentinian vegetarians and non-vegetarians, according to the guidelines for healthful vegetarian diets [47]. We also explored some sociodemographic factors, self-defined proportion of household income spent on food, and the accessibility of vegetarian foods.

## 2. Materials and Methods

### 2.1. Study Population

Individuals of 18 years and older were invited to participate in the study mainly through social networks. The invitation was addressed to those interested in knowing the adequacy of their health-related lifestyle habits, and interested in receiving an immediate and personalized feedback based on the major recommendations of the guidelines for healthful vegetarian diets and lifestyle proposed by the Department of Nutrition, School of Public Health at Loma Linda University [47]. Participants from the 23 Argentinian states were enrolled in the study, as well as some who lived abroad. We excluded participants who did not adequately complete the questions of the study.

### 2.2. Ethical Aspects

A written informed consent was provided at the first electronic page containing the invitation to join in the study. Participants were enrolled after clicking the “accept” icon, meaning that they have agreed with the informed consent terms. All procedures associated with this project were conducted following the international ethical standards proposed by the Helsinki protocol for human research and this study was reviewed and approved by the Research and Ethics Committee of the Adventist University of River Plate School of Medicine (resolution #12/8-2018). This committee is affiliated to the National Register of Health Research (registered under the #000237) of the Ministry of Health, Argentine.

### 2.3. Sociodemographic Information, Dietary and Lifestyle Factors

Sociodemographic and health information, as well as lifestyle and dietary data were collected using an online survey available to be self-administered through the web link (Supplementary Materials). Briefly, participants provided information on age, city and state of residence, gender, education (elementary, secondary school, tertiary/college and university), employment status, and type of occupation. Data collected also included, cigarette smoking (including numbers of cigarettes), alcohol consumption (frequency of intake), body weight, and height. The history of a medical condition diagnosed by a physician, such as arterial hypertension, cardiovascular diseases, dyslipidemia, diabetes, cancer, chronic respiratory diseases, osteomuscular conditions, thyroid dysfunctions, celiac disease, rheumatoid arthritis, depressive disorders, and other chronic health conditions.

The dietary pattern was self-reported and classified according to Dagnelie & Mariotti [48]. Briefly, non-vegetarians consume red meats, poultry and/or fish regularly; semi-vegetarians consume red meats, poultry or fish no more than once a week; pesco-vegetarians consume fish but not red meat or poultry; lacto-ovo-vegetarians consume eggs and dairy products but not meats; and vegans do not consume meats, dairy products or eggs. The time of adherence to the current dietary habits was classified as less than 6 months, 6 to 12 months, and more than 12 months.

The adherence to healthy lifestyle habits was assessed based on the criteria proposed by the Vegetarian Lifestyle Index [7], according to the main recommendations of the guidelines for healthful vegetarian diets and lifestyle proposed by the Department of Nutrition, School of Public Health, Loma Linda University [47]. Fourteen items were considered, with eleven related to diet and three related to lifestyle habits. Diet components encompassed a whole-food plant-based nutrition including the consumption of whole grains, legumes and soy, vegetables, fruits, nuts and seeds, dairy products, eggs, sweets, reliable sources of vitamin B-12, and meat foods. The following lifestyle habits were also considered: daily exercise, water intake, and moderate skin exposure to sunlight. Each item presented 3 options of answers and scored 0, 0.5, or 1 point. A score value of 1 was attributed when participants referred to consume ≥6 servings/day of whole grains, ≥3 servings/day of legumes, ≥8 servings/day of vegetables, ≥4 servings/day of fruits, ≥1.5 servings/day of nuts and seeds, 0–2 servings/day of vegetables oils, 0 servings/day of dairy products and eggs, 0–2 servings/week of sweet, ≥2.0 mcg serving equivalent/day of vitamin B-12, 0 servings/day of meat foods, ≥8 glasses water/day, and ≥30 min/day of moderate physical activity or ≥15 min/day of vigorous physical activity, and ≥10 min/day of sunlight skin exposure between 11 a.m. and 1 p.m. The selected cut-off points were defined based on the recommendations of a diet containing approximately 2000 kcal/day for vegans [47]. The cut-offs points of all items are presented in Table 3. Components were equally weighted and the sum of them generates a composite score ranging from 0 to 14 points. Higher total scores indicate greater adherence to healthy vegan lifestyle habits.

### 2.4. Online Resources and Feedback to Participants

After completing the online survey, participants received an immediate and personalized feedback. For each question, two types of motivational feedback could be offered, depending on the participant’s answer. If the answer was associated with a score 0 or 0.5 points, participants were encouraged to ‘put effort’ to achieve a healthier habit associated with the lifestyle factor which scored low. On the other hand, if the answer scored 1 point, a ‘keep going’ message was delivered (Supplementary Materials). Questions were designed in the Survey Monkey online platform using the Premier plan (SurveyMonkey, San Mateo, CA, USA). Each question included an illustrated image obtained from Shutterstock (New York, NY, USA) used to help participants to choose food portions. To generate the personalized feedback to each one of the 14 answers, images containing an explanatory text and a motivational figure were designed in JPG format. Based on the participant’s answer, a specific image was selected from a set of Cloud Functions of Google Firebase-Blaze plan (Google, San Francisco, CA, USA). The total composite score (gauge type image) was generated using the Application Programming Interface (API) of Google Image Charts (Google, San Francisco, CA, USA), and shows the total percentage obtained by the participant over one hundred percent based on the fourteen points evaluated. Participants could also choose to receive an e-mail copy of their feedback using the SendGrid’s Free Plan API (SendGrid, Denver, CO, USA). All images, graphics, and vectors were used with previous permission or were under a “Creative Commons 0” license.

### 2.5. Statistical Analysis

Descriptive analyses were carried out for sociodemographic, lifestyle, health and dietary information. Chi-square test was used for assessing differences in categorical variables between groups. The score of adherence to healthy vegan lifestyle habits was compared according to selected demographic variables. To compare the scores among different dietary patterns, we used the Mann–Whitney *U*-test and the Kruskal–Wallis. Analysis of variance (ANOVA) was conducted to obtain descriptive statistics and unadjusted mean scores according to demographic and lifestyle characteristics of the population. We used a modeling approach to determine mean scores across dietary patterns, with the dietary pattern as the independent variable and the adherence score as the dependent variable in Model 1. Analysis of covariance (ANCOVA) was performed to compare the adjusted means of the adherence scores by categories of the dietary pattern at 95% confidence interval (95% CI). Model 2 was adjusted for gender and age. Model 3 was adjusted as in Model 2 plus body mass index (BMI), tobacco and current alcohol use. Model 4 was adjusted as in Model 3 plus educational level. All statistical analyses were performed using the software SPSS version 22 (SPSS Inc., Chicago, IL, USA). *p*-values < 0.05 were considered statistically significant.

## 3. Results

A total of 1454 participants completed the study. Regarding sociodemographic and other selected characteristics shown in Table 1, most of the participants were females (84.9%), young-adults (mean age 32.1, SD = 13.6, 18–82 years), and single (55.0%). Approximately one half of the population had with high educational level (50.4%) and was employed (50.8%). The population was comprised of individuals from all states of Argentina and from 6 main regions of the country: Northeast, Northwest, Central, Cuyo, Buenos Aires, and South. A small number of participants (2.5%) informed that they were currently living out of Argentina. Between 7 and 8% of participants were tobacco cigarette smokers and referred to consume alcohol regularly (more than once a week). Almost half of the participants were overweight or obese (45.6%), and 33.1% had a medical diagnosis of a chronic condition according to self-reported anthropometric and health information. The dietary patterns were represented by two main groups of non-vegetarians (29.5%), and vegetarians (70.5%). Lacto-ovo-vegetarians were the most prevalent group among vegetarians, and 146 (10%) individuals were vegans. More than 2/3 of the study population referred to adhere to the current dietary pattern for more than 12 months. The mean score of adherence to a healthy vegan lifestyle for the study population was 6.64, SD = 1.72. Table 1 also presents the score of adherence to healthy vegan lifestyle habits in relation to sociodemographic and other characteristic, showing no significant differences for gender, educational level, marital and occupational status, regions of Argentina, time of adherence to dietary pattern and the presence of chronic diseases. The score of adherence to healthy vegan lifestyle habits varied significantly among individuals in different age groups, with distinct smoking status, alcohol use, BMI, and dietary patterns.

Table 2 shows that males tend prefer meat-containing diet patterns, such as the non- and semi-vegetarian patterns. Pesco-vegetarian and vegan dietary patterns showed higher proportions of females. Adults between 21 and 64 years old were mostly non-vegetarians. Vegetarian dietary patterns (semi-vegetarians, pesco-vegetarians, lacto-ovo-vegetarians, and vegans) were more prevalent at younger ages and among older individuals. There was a higher prevalence of vegetarian dietary patterns among individuals who were currently married, married in the past, retired, living in Buenos Aires and central areas of Argentina, non-tobacco smokers, non-alcohol users, and with lower BMI (underweight and normal weight). Females, never married, employed, living in Buenos Aires, non-smoker, non-alcohol user, normal and underweight BMI, without any chronic conditions were the most prevalent characteristics among vegans. There were no significant associations between the dietary patterns, educational levels and smoking status. The presence of one or more chronic condition was more prevalent in non-vegetarians and semi-vegetarians compared with pesco-vegetarians, lacto-ovo-vegetarians, and vegans. Significant differences were found considering the proportion of the total household income spent on food among all dietary patterns. The mean proportion of household income spent on food was significantly lower among vegans when compared with other dietary patterns.

According to the data shown in Table 3, the study population presented a low consumption of whole grains, legumes, vegetables, nuts, and seeds. A high intake of vegetable oils and an intermediate consumption of fruits, dairy products, eggs, reliable sources of vitamin B-12, and water were detected. Sunlight exposure was low or moderate in most individuals. Moreover, participants presented low levels of regular physical activity practice.

Considering the recommended intake of different food groups, we found that a vegan dietary pattern was significantly associated with the consumption of ≥6 servings/day of whole grains, ≥3 servings/day of legumes, ≥4 servings/day of fruits, ≥1.5 servings/day nuts and seeds. The consumption of ≥8 servings/day of vegetables, 0–2 servings/day of vegetable oils, and 0–2 servings/week of sweets was associated with a pesco-vegetarian pattern. The intake of >2 servings/day of dairy, and >1 servings/day of eggs was associated with the non-vegetarian dietary pattern. The intake of sweets <2 servings/week were similarly associated with the pesco-vegetarian and vegan diets. The vegan dietary pattern was associated with the lowest intake of vitamin B-12 (<1.0 mcg/serving equivalent day). There was no association of dietary patterns with daily exercise, water intake, and sunlight exposure in this population.

Table 4 reports the adjusted and unadjusted mean scores of adherence to healthy vegan lifestyle habits by categories of vegetarian dietary patterns. Non-vegetarians had a significantly lower mean score (5.72; 95% CI, 5.58–5.85) than the vegetarian groups, including semi- (6.33; 95% CI 6.18–6.48), pesco- (6.97; 95% CI, 6.57–7.36), and lacto-ovo-vegetarians (7.11; 95% CI, 6.96–7.27), as well as vegans (8.62; 95% CI, 8.37–8.87). Some minor changes occurred after adjusting for sociodemographic and lifestyle factors in Model 4, meaning that non-vegetarians (5.75; 95% CI, 5.61–5.89) had significantly lower mean compared to semi- (6.32; 95% CI, 6.17–6.47), pesco- (6.99; 95% CI, 6.59–7.39), lacto-ovo-vegetarians (7.10; 95% CI, 6.96–7.24), and vegans (8.59; 95% CI, 8.35–8.83).

Table 5 shows the vegetarian food availability for purchase nearby the participant’s neighborhood. Most of the participants reported buying vegetarian foods in grocery stores with a distance of less than 1 km from their residence. Natural fruit juices, fortified dairy milk, nuts, seeds, and olive oils are the most available foods. Tofu and other vegan cheeses are the least bought foods by this population.

## 4. Discussion

This study provides new data about healthy vegan lifestyle habits, sociodemographic and other selected characteristics among non-vegetarians and vegetarians in Argentina. Research about vegetarian nutrition and its influence on health are scarce in South America, and especially in Argentina where meat food intakes are high [49,50]. In our study, vegetarians showed healthier lifestyle habits and lower risk factors for NCDs than non-vegetarians. However, levels of adherence to whole-food plant-based diets are insufficient including for vegans. Another finding is that the mean score composite of adherence to a healthy vegan lifestyle was significantly different among the dietary patterns evaluated. The higher mean score of adherence to a healthy vegan lifestyle was found in the vegan dietary pattern, followed by the lacto-ovo-vegetarian, pesco-vegetarian, semi-vegetarian, and non-vegetarian dietary pattern, even after adjusting for sociodemographic characteristics and lifestyle factors. The approaches used in our study could discriminate the eating habits of different dietary patterns as similar to other research done with vegetarians [7,51].

The predominant group of participants of this study was comprised of highly educate young adult females, as observed in other investigations of vegetarian populations [13,51,52,53,54]. There were no significant differences in the mean score of adherence to a healthy vegan lifestyle among categories of sociodemographic variables such as gender, educational level, marital and occupational status, and region of residencies in Argentina, suggesting certain homogeneity in the studied population concerning to diet choices. In contrast, the mean score of adherence to a healthy vegan lifestyle was significantly higher in non-smokers, non-alcohol consumers, and individuals with normal BMI, showing the association of higher lifestyle scores with important protective factors for NCDs. Most of the participants reported having had the current dietary pattern for more than 12 months suggesting that the information provided reflects a certain adherence to their current dietary pattern.

The participation of individuals from different areas of Argentina reveals the wide distribution of the online survey of this study throughout the country. Some regions had higher participation such as the capital zone, Buenos Aires. The study was also announced during the VegFest-Argentina, a meeting held in this city, reaching out individuals from different parts of the country interested in vegetarian diets. This explains the expressive number of vegan participants. We also advertised this research through the social networks of our University in the state of Entre Ríos, a central area of Argentina, influenced by the immigration of Russo-German Adventists [55] involved with the promotion of healthy lifestyles, including vegetarian diets. Some of the findings of this study such as the low prevalence of tobacco cigarette smoking, and alcohol intake, besides the high prevalence of vegetarian dietary patterns, with relation to the general population of Argentina, suggest that participants of this study show characteristics of being a health/vegetarian-oriented population.

Several studies associate healthy vegetarian lifestyles with adequate body weight and better health status [13,56]. The BMI information from this study showed a lower prevalence of overweight and obesity when compared to similar self-reported statistics of the general Argentinian population [57,58]. This may be attributed to the high prevalence of individuals with vegetarian dietary patterns in this population, similarly found in other research [38,59,60]. In line with this, our study also reported a lower prevalence of chronic conditions throughout all dietary patterns but particularly in vegetarians as found in similar studies [13,20,61].

In our study, participants have self-defined their dietary patterns. This classification was mostly corroborated with the indicated consumption of different food groups. For instance, vegans did not consume meat or flesh products. However, around 5% of this population reported some occasional intake of dairy/eggs. The same proportion of lacto-ovo-vegetarians also reported occasional meat intake (Table 3). This may depend on limit of self-reported classification of participants.

Individuals following the vegan dietary pattern had highest intakes of whole grains, legumes, fruits, and nuts and seeds compared with other dietary groups. Pesco-vegetarians and lacto-ovo-vegetarians showed to some extent similar prevalence of consumption of legumes, vegetables, fruits, nuts and seeds. The lowest intakes of sweets were found similarly in vegans and pesco-vegetarians. However, it should be highlighted that a global analysis of the results obtained from all participants, including vegans, show the need of improvements in dietary and lifestyle habits. Some of food groups, including whole grains, legumes, vegetables, nuts, and seeds are not properly consumed by the general population of this study. Only around 5% of the total population showed adequate intakes of legumes and vegetables according to the standards proposed for a healthy vegan diet. Also, a very low intake of whole grains was observed, suggesting that this population may be consuming predominantly refined-processed grains. Unhealthy dietary and lifestyle habits may be associated with the high prevalence of NCDs observed in Argentina [49,58]. Several studies show that adequate intakes of fruits and vegetables are associated with lower morbidity and mortality for NCDs [35,50,62,63]. Corroborating with this, we have previously described a very low intake of fruits and vegetables in the general population of some areas of Argentina associated with high prevalence of risk factors for NCDs, and multimorbidity, disproportionately affecting young adults [43]. We also found low consumption of fruits, vegetables, and legumes in men with prostate cancer and other cancers in Argentina [64].

When following a vegan dietary pattern, it is important to get appropriate amounts of a variety of whole-foods to obtain macro- and micronutrients to achieve nutritional adequacy. In our study, the vegan dietary pattern reported the lowest prevalence of intake of reliable sources of vitamin B-12. Studies show that persons adopting a vegan dietary pattern should regularly consume vitamin B-12 fortified foods or take vitamin B-12 supplements [65,66].

Other health-related lifestyle habits, such as sunlight exposure and physical activity, were examined and found to be similarly adopted among different dietary pattern groups. The sunlight exposure was either low or intermediate in all groups including in vegans, therefore the organic supply of vitamin D may be impaired, unless proper replacement of vitamin D is provided with fortified foods or supplements [67,68,69]. Studies show that vitamin D produced through sunlight exposure has a longer half-life than of those obtained from other sources, and helps to maintain serum vitamin D concentrations within the normal range [70,71].

A meat-free diet is not enough to guarantee a healthy lifestyle. Despite the known health benefits of vegetarian diets [65,72], other aspects of the lifestyle such as physical activity are also important for health promotion and prevention of NCDs [73,74,75]. In the present study the use of tobacco and alcohol intake was lower in the vegetarian dietary patterns compared with non-vegetarians showing the consistency regarding the adoption of healthy lifestyle habits by vegetarians as similarly shown in some studies with vegetarians, including vegans [21,76,77]. In a recent investigation, vegans do not differ much from non-vegetarians in regard to non-eating health related behaviors such as tobacco smoking, alcohol intake and physical activity [40]. Thus, it is important to discriminate between vegetarian diets from healthy dietary and lifestyle habits.

The score of adherence to a healthy vegan lifestyle of our study was based on the dietary and the lifestyle components of the Vegetarian Lifestyle Index [7], using an online survey, and attributing higher scores for increasing adherence to healthful vegan diet and lifestyle factors [7,47]. The score of adherence to a healthy vegan lifestyle calculated in our study was based on the recommendations for an approximately 2000 kcal vegan diet according to the recommendations from the food pyramid guide from the Loma Linda University [47]. For practical reasons, the components to measure the adherence to a healthy vegan lifestyle were presented in an online survey as a simple tool to an initial assessment of main health-related lifestyle habits. Several studies mention that it is opportune to use online methods for carrying out health surveys, as they are reliable, fast, effective, and interactive, avoiding some of the barriers presented in traditional approaches [78,79].

Moreover, this e-health survey may be a helpful tool for primary health care contexts for health promotion and prevention of NCDs. The healthy vegan lifestyle survey also has some educational purposes since the feedbacks may help to trigger the interest of participants to implement lifestyle changes. This form of personalized feedback derived from the individual evaluation has been shown to be valuable in other studies [79]. We speculate that several persons were attracted to participate in the study in order to receive an immediate feedback about their dietary/lifestyle current status. It is interesting to observe that an important number of non-vegetarians also participated in the study, which may suggest a concern about issues associated with diet and lifestyle.

Regarding the score of adherence to a healthy vegan lifestyle, the findings of our study point to a steady increase in the mean score from (5.72, SD = 1.41) in non-vegetarians to (8.62, SD = 1.52) in vegans. This variability is consistent with supplanting of animal products and adopting whole food plant-based nutrition. It would be important that future prospective studies to be developed in South America correlate vegetarian lifestyle scores with different health outcomes since vegans in other studies were shown to present higher scores for dietary components such as in ours [51,80]. The mean score of the adherence to a healthy vegan lifestyle in this Argentinian population (6.64, SD = 1.72) is lower than the score (7.42, SD = 1.75) found in the Adventist Health Study-2 cohort investigation considering the recommendations for a 2000 kcal for lacto-ovo-vegetarian diet with moderate intakes of dairy and eggs [21]. Although both populations presented health-oriented profiles, several factors may have accounted for the observed differences as of diets considered, socioeconomic factors, religious and cultural idiosyncrasies.

In this study, we also evaluated the accessibility of vegetarian foods and we find that it is possible for participants to find healthy vegan foods within a relatively nearby area of the neighborhood. It should be noted that the vegan foods’ availability reported by consumers is higher for natural foods than for processed food products. This is especially important since the healthy plant-based diet incorporate more whole grains, vegetables, fruits, seeds and nuts in a natural form or in some simple-homemade preparations [50,81]. Processed foods tend to incorporate more salt, sugar, and chemical additives which may compromise their nutritional value and health effects [39,82,83]. In Argentina, vegetarian dietary patterns are not very common and according to the participants of this study, the processed food options for vegans are scarce but this may be an advantage to develop a healthier vegan lifestyle.

## 5. Conclusion

The major findings of our study indicate that vegetarians in Argentina have healthier lifestyle habits, with lower risk factors for NCDs than non-vegetarians independent of their sociodemographic characteristics. In addition, individuals adopting the vegan dietary pattern presented the highest mean score of adherence to a healthy vegan lifestyle, lower prevalence of unhealthy habits (i.e., tobacco smoking, alcohol intake), and lower BMI compared to the individuals with other dietary patterns. However, it should be noted that the promotion of nutritional health education merits further attention and needs to be encouraged in all dietary groups to increase the compliance with recommendations for healthy diets and improved lifestyle habits in order to address risk factors associated with NCDs in Argentina.

## Figures and Tables

**Table 1:** Distribution of the score of adherence to healthy vegan lifestyle habits among individuals from an Argentinian population according to sociodemographic and other selected characteristics.

Characteristics	Distribution		Score of Adherence to HVLH		
	*n*	(%)	Mean	SD	*p*-Value for Kruskal–Wallis
Gender					NS *
Female	1.234	(84.9)	6.60	1.70	
Male	220	(15.1)	6.85	1.83	
Age					0.025
18–20 years	271	(18.6)	6.80	1.71	
21–64 years	1.132	(77.9)	6.58	1.73	
>64 years	51	(3.5)	7.05	1.43	
Education level					NS
Secondary school or less	420	(28.9)	6.55	1.69	
Tertiary	301	(20.7)	6.54	1.77	
University	612	(42.1)	6.68	1.68	
Graduate school	121	(8.3)	6.95	1.88	
Marital status					NS
Never married	800	(55.0)	6.66	1.70	
Currently married	574	(39.5)	6.63	1.78	
Married in the past	80	(5.5)	6.44	1.54	
Occupational status					NS
Student	489	(33.6)	6.70	1.73	
Health professional	69	(4.7)	6.82	1.72	
No health professional	671	(46.1)	6.62	1.74	
Unpaid domestic work	88	(6.1)	6.25	1.65	
Retired	77	(5.3)	6.92	1.62	
Unemployed	55	(3.8)	6.21	1.46	
Region of Argentina					NS
Northeast	112	(7.7)	6.63	1.78	
Northwest	58	(4.0)	6.27	1.87	
Central	652	(44.8)	6.76	1.66	
Cuyo region	93	(6.4)	6.32	1.68	
Buenos Aires region	405	(27.9)	6.56	1.78	
South	98	(6.7)	6.53	1.50	
Out of Argentina **	36	(2.5)	7.02	2.10	
Smoking status					0.041
Non-smoker	1.343	(92.4)	6.67	1.70	
Up to 10 cigarettes/day	94	(6.5)	6.25	1.89	
More than 10 cigarettes/day	17	(1.2)	6.02	1.84	
Alcohol use					<0.001
Never	792	(54.5)	6.80	1.76	
Up to once a week	552	(38.0)	6.51	1.69	
More than once a week	110	(7.6)	6.09	1.44	
BMI					<0.001
Underweight	67	(4.6)	6.52	1.78	
Normal weight	866	(59.6)	6.85	1.74	
Overweight	345	(23.7)	6.46	1.60	
Obese	173	(11.9)	5.97	1.63	
NCD					NS
No	983	(67.6)	6.68	1.75	
One condition	407	(28.0)	6.60	1.62	
Multimorbidities	64	(4.4)	6.21	1.86	
Vegetarian dietary pattern					<0.001
Non-vegetarian	429	(29.5)	5.72	1.41	
Semi-vegetarian	393	(27.0)	6.33	1.48	
Pesco-vegetarian	52	(3.6)	6.97	1.40	
Lacto-ovo-vegetarian	434	(29.8)	7.11	1.59	
Vegan	146	(10.0)	8.62	1.52	
Time of adherence to current dietary pattern					NS
Less than 6 months	168	(11.6)	6.76	1.60	
6–12 months	196	(13.5)	6.80	1.66	
More than 12 months	1090	(75.0)	6.59	1.75	

HVLH = Healthy Vegan Lifestyle Habits; NS = Non-significant; BMI = body mass index; NCD = non-communicable diseases; SD = standard deviation; * *p*-value for Mann–Whitney *U*-test; ** Argentinian living out of Argentina.

**Table 2:** Sociodemographic and other selected characteristics according to the vegetarian dietary pattern in an Argentinian population.

Characteristics	Non-		Semi-		Pesco-		Lacto-ovo		Vegan		*p*-Value *
	*n*	%	*n*	%	*n*	%	*n*	%	*n*	%	
Gender											0.009
Female	348	28.2	330	26.7	50	4.1	375	30.4	131	10.6	
Male	81	36.8	63	28.6	2	0.9	59	26.8	15	6.8	
Age											0.001
18–20 years	72	26.6	69	25.5	9	3.3	76	28.0	45	16.6	
21–64 years	348	30.7	303	26.8	39	3.4	344	30.4	98	8.7	
>64 years	9	17.6	21	41.2	4	7.8	14	27.5	3	5.9	
Education level											NS
High school or less	120	28.6	105	25.0	17	4.0	132	31.4	46	11.0	
Tertiary	93	30.9	90	29.9	12	4.0	73	24.3	33	11.0	
University	180	29.4	166	27.1	19	3.1	195	31.9	52	8.5	
Graduate school	36	29.8	32	26.4	4	33	34	28.1	15	12.4	
Marital status											0.033
Never married	241	30.1	188	23.5	34	4.3	252	31.5	85	10.6	
Currently married	166	28.9	175	30.5	14	2.4	164	28.6	55	9.6	
Married in the past	22	27.5	30	37.5	4	5.0	18	22.5	6	7.5	
Occupational status											0.003
Student	132	27.0	122	24.9	22	4.5	163	33.3	50	10.2	
Health professional	27	39.1	13	18.8	2	2.9	21	30.4	6	8.7	
No health professional	195	29.1	192	28.6	19	2.8	197	29.4	68	10.1	
Unpaid domestic work	33	37.5	29	33.0	4	4.5	16	18.2	6	6.8	
Retired	14	18.2	30	39.0	5	6.5	20	26.0	8	10.4	
Unemployed	26	47.3	6	10.9	0	0.0	16	29.1	7	12.7	
Region of Argentina											<0.001
Northeast	48	42.9	36	32.1	2	1.8	15	13.4	11	9.8	
Northwest	26	44.8	7	12.1	1	1.7	17	29.3	7	12.1	
Central	170	26.1	198	30.4	23	3.5	223	34.2	38	5.8	
Cuyo	35	37.6	19	20.4	2	2.2	27	29.0	10	10.8	
Buenos Aires	112	27.7	85	21.0	18	4.4	124	30.6	66	16.3	
South	33	33.7	33	36.7	4	4.1	20	20.4	8	8.2	
Out of Argentina ^†^	5	13.9	15	41.7	2	5.6	8	22.2	6	16.7	
Smoking status											NS
Non-smoker	383	28.5	370	27.6	48	3.6	403	30.0	139	10.3	
Up to 10 cigarettes/day	39	41.5	18	19.1	3	3.2	27	28.7	7	7.4	
>10 cigarettes/day	7	41.2	5	29.4	1	5.9	4	23.5	0	0.0	
Alcohol use											<0.001
Never	181	22.9	248	31.3	27	3.4	252	31.8	84	10.6	
Up to once a week	195	35.3	122	22.1	21	3.8	158	28.6	56	10.1	
More than once a week	53	48.2	23	20.9	4	3.6	24	21.8	6	5.5	
BMI											<0.001
Underweight	16	23.9	18	26.9	6	9.0	16	23.9	11	16.4	
Normal weight	232	26.8	212	24.5	36	4.2	280	32.3	106	12.2	
Overweight	109	31.7	114	33.0	4	1.2	100	29.0	18	5.2	
Obese	71	41.0	49	28.3	6	3.5	37	21.4	10	5.8	
NCD											0.015
No	283	28.8	242	24.6	35	3.6	314	31.9	109	11.1	
One condition	129	31.7	127	31.2	17	4.2	102	25.1	32	7.9	
Multimorbidities	17	26.6	24	37.7	0	0.0	18	28.1	5	7.8	
Household income spent on food (mean; SD)	45.3	20.4	41.6	19.2	42.4	16.1	40.2	18.7	38.6	19.2	0.001 ^¥^

NS = Non-significant; BMI = body mass index; NCD = non-communicable disease; SD = standard deviation, * *p-value* for chi-square test; ^†^ Argentinian living out of Argentina; ^¥^ *p-value* for Kruskal-Wallis test.

**Table 3:** Habits related to healthy vegan diet and lifestyle according to vegetarian dietary pattern in an Argentinian population.

Vegan Lifestyle Habits		Score	Total		Non-		Semi-		Pesco-		Lacto-Ovo		Vegan		*p*-Value *
			*n*	%	*n*	%	*n*	%	*n*	%	*n*	%	*n*	%	
**Food groups**															
Whole grains	<3 servings/day	0	920	63.3	315	73.4	261	66.4	35	67.3	246	56.7	63	43.2	<0.001
	≥3 and <6 servings/day	0.5	485	33.4	104	24.2	123	31.3	15	28.8	170	39.2	73	50.0	
	≥6 servings/day	1	49	3.4	10	2.3	9	2.3	2	3.8	18	4.1	10	6.8	
Legumes, soy and meat substitutes	<1 serving/day	0	693	47.7	309	72.0	211	53.7	21	40.4	135	31.1	17	11.6	<0.001
	≥1 and <3 servings/day	0.5	677	46.6	114	26.6	173	44.0	27	51.9	261	60.1	102	69.9	
	≥3 servings/day	1	84	5.8	6	1.4	9	2.3	4	7.7	38	8.8	27	18.5	
Vegetables	<4 servings/day	0	846	58.2	278	64.8	238	60.6	27	51.9	233	53.7	70	47.9	0.006
	≥4 and <8 servings/day	0.5	537	36.9	137	31.9	133	33.8	21	40.4	179	41.2	67	45.9	
	≥8 servings/day	1	71	4.9	14	3.3	22	5.6	4	7.7	22	5.1	9	6.2	
Fruits	<2 servings/day	0	532	40.0	204	47.6	144	36.6	16	30.8	174	40.1	44	30.1	<0.001
	≥2 and <4 servings/day	0.5	703	48.3	185	43.1	208	52.9	30	57.7	209	48.2	71	48.6	
	≥4 servings/day	1	169	11.6	40	9.3	41	10.4	6	11.5	51	11.8	31	21.2	
Nuts and seeds	<4 servings/week	0	767	52.8	271	63.2	216	55.0	18	34.6	218	50.2	44	30.1	<0.001
	≥4 servings/week and <1.5 servings/day	0.5	440	30.3	117	27.3	113	28.8	20	38.5	131	30.2	59	40.4	
	≥1.5 servings/day	1	247	17	41	9.6	64	16.3	14	26.9	85	19.6	43	29.5	
Vegetable oils	>4 servings/day	0	35	2.4	10	2.3	10	2.5	1	1.9	8	1.8	6	4.1	NS
	>2 and ≤4 servings/day	0.5	292	20.1	90	21.0	75	19.1	6	11.5	85	19.6	36	24.7	
	≤2 servings/day	1	1127	77.5	329	76.7	308	78.4	45	86.5	341	78.6	104	71.2	
Dairy products	>2 servings/day	0	291	20	126	29.4	76	19.3	12	23.1	77	17.7	0	0.0	<0.001
	>0 and ≤2 servings/day	0.5	789	54.3	249	58.0	250	63.6	29	55.8	253	58.3	8	5.5	
	0 serving/day	1	374	25.7	54	12.6	67	17.0	11	21.2	104	24.0	138	94.5	
Eggs	>1 serving/day	0	238	16.4	95	22.1	57	14.5	8	15.4	78	18.0	0	0.0	<0.001
	>0 and ≤1 serving/day	0.5	927	63.8	288	67.1	295	75.1	41	78.8	296	68.2	7	4.8	
	0 serving/day	1	289	16.4	46	10.7	41	10.4	3	5.8	60	13.8	139	95.2	
Sweets	>5 servings/week	0	215	14.8	89	20.7	44	11.2	8	15.4	59	13.6	15	10.3	<0.001
	>2 and ≤5 servings/week	0.5	605	41.6	190	44.3	179	45.5	14	26.9	174	40.1	48	32.9	
	0–2 servings/week	1	634	43.6	150	35.0	170	43.3	30	57.7	201	46.3	83	56.8	
Reliable sources of vitamin B-12	<1.0 mcg serving equivalent/day	0	394	27.1	26	6.1	102	26.0	19	36.5	159	36.6	88	60.3	<0.001
	≥1.0 and <2.0 mcg serving equivalent/day	0.5	604	41.5	162	37.8	190	48.3	22	42.3	185	42.6	45	30.8	
	≥2.0 mcg serving equivalent/day	1	456	31.4	241	56.2	101	25.7	11	21.2	90	20.7	13	8.9	
Flesh-food intake	>1 time/week	0	545	37.5	400	93.2	130	33.1	8	15.4	7	1.6	0	0.0	<0.001
	>1 time/month and ≤1 time/week	0.5	291	20.0	27	6.3	230	58.5	16	30.8	18	4.1	0	0.0	
	≤1 time/month	1	618	42.5	2	0.5	33	8.4	28	53.8	409	94.2	146	100	
**Other lifestyle components**															
Daily exercise	0 min/day of any moderate or vigorous exercise	0	261	18.0	92	21.4	66	16.8	7	13.5	77	17.7	19	13.0	NS
	>0 and <30 min/day of moderate or >0 and <15 min/day of vigorous exercise	0.5	536	36.9	137	31.9	150	38.2	18	34.6	166	38.2	65	44.5	
	≥30 min/day of moderate or ≥15 min/day of vigorous exercise	1	657	45.2	200	46.6	177	45.0	27	51.9	191	44.0	62	42.5	
Water intake	<4 glasses of water/day	0	309	21.3	98	22.8	88	22.4	11	21.2	96	22.1	16	11.0	NS
	≥4 and <8 glasses of water/day	0.5	702	48.3	200	46.6	185	47.1	27	51.9	214	49.3	76	52.1	
	≥8 glasses of water/day	1	443	30.5	131	30.5	120	30.5	14	26.9	124	28.6	54	37.0	
Sunlight exposure	<5 min/day	0	533	36.7	163	38.0	140	35.6	20	38.5	168	38.7	42	28.8	NS
	≥5 and <10 min/day	0.5	555	38.2	152	35.4	154	39.2	23	44.2	161	37.1	65	44.5	
	≥10 min/day	1	366	25.2	114	26.6	99	25.2	9	17.3	105	24.2	39	26.7	

NS = Non-significant; * *p-value* for chi-square test.

**Table 4:** Mean score of adherence to heathy vegan lifestyle habits according to vegetarian dietary patterns in an Argentinian population.

Vegetarian Dietary Pattern	Model 1			Model 2		Model 3		Model 4	
	Mean	±SD	95% CI	Mean	95% CI	Mean	95% CI	Mean	95% CI
Non-vegetarian	5.72	1.41	5.58–5.85	5.70	5.56–5.84	5.75	5.61–5.89	5.75	5.61–5.89
Semi-vegetarian	6.33	1.48	6.18–6.48	6.33	6.18–6.47	6.32	6.18–6.47	6.32	6.17–6.47
Pesco-vegetarian	6.97	1.40	6.57–7.36	7.02	6.61–7.42	6.98	6.57–7.38	6.99	6.59–7.39
Lacto-ovo-vegetarian	7.11	1.59	6.96–7.27	7.12	6.98–7.26	7.10	6.96–7.24	7.10	6.96–7.24
Vegan	8.62	1.52	8.37–8.87	8.64	8.40–8.88	8.58	8.34–8.83	8.59	8.35–8.83

Model 1: Unadjusted model. Model 2: Adjusted for gender and age. Model 3: Adjusted as in Model 2 + BMI, tobacco and alcohol use. Model 4: Adjusted as in Model 3 + educational level. Marginal (adjusted) means were reported for analysis of covariance (ANCOVA) models 2, 3, and 4. A global *p*-trend test with *p* < 0.05 indicates statistical significance. Model 1, 2, 3, and 4 (*p*-trend < 0.0001); CI = confidence interval.

**Table 5:** Vegetarian food availability referred by consumers from an Argentinian population.

Food Groups and Products	Less Than 1 km		More Than 1 km		Internet		Total Availability ^†^	
	*n*	%	*n*	%	*n*	%	*n*	%
Fortified dairy milk and yogurt *	761	70.5	296	27.4	13	1.2	969	89.7
Vegetable (milk) drink	488	33.5	311	21.4	11	0.8	779	53.6
Tofu and other vegan cheeses	276	19.0	197	13.5	16	1.1	453	31.8
Vegan hamburgers	517	35.6	276	19.0	37	2.5	760	52.3
Vegetable cold cuts	325	22.4	218	15.0	27	1.9	543	37.3
Vegan bread and desserts	531	36.5	313	21.5	38	2.6	814	56.0
100% natural fruit juices	576	39.5	235	16.2	15	1.0	1454	100.0
Olive oil	795	54.7	432	29.7	25	1.7	1189	81.8
Seeds	840	57.8	505	34.7	21	1.4	1276	87.8
Nuts	803	55.2	533	36.7	51	3.5	1288	88.6

* Frequencies considered only for the dairy milk consumers. ^†^ These values for the total availability show the frequencies of participants who refer to buy foods or food products in at least one place of purchase. Others may be either non-consumers of the products or have indicated its unavailability.

## Supplementary Materials

The following are available online at http://www.mdpi.com/2072-6643/11/1/154/s1: The online survey with a schematic representation of the electronic feedback provided to participants with the total composite score.
