# Supplementary material for: Healthy Lifestyle Practices among Argentinian Vegetarians and Non-Vegetarians
Source: Nutrients. 2019 Jan 12;11(1):154. doi: 10.3390/nu11010154 (PMC6356967; doi:10.3390/nu11010154)
Supplement: Supplementary file 1 [file nutrients-11-00154-s001.pdf]

INDICE DE

Calidad de dieta Vegetariana

¡Bienvenido!

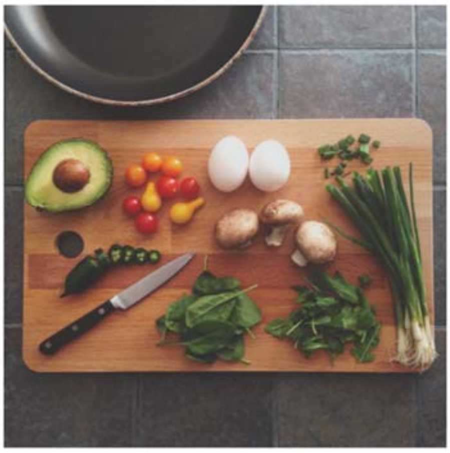

Estimado Participante:

Usted está invitado a participar de una encuesta en línea sobre un Índice de calidad de dieta vegetariana* desarrollado por la Universidad de Loma Linda en California, USA, y adaptado por investigadores argentinos.

Al final de la encuesta, usted recibirá una devolución informándole el porcentaje obtenido de este índice y sugerencias para mejorar su alimentación acorde a sus respuestas. Esto no reemplaza, de ninguna manera, evaluaciones y tratamientos realizados por profesionales de salud.

Su participación es totalmente voluntaria y anónima, con una duración aproximada de 15 minutos. Usted puede negarse a participar o salir de la encuesta en cualquier momento. No recibirá ningún tipo de beneficio económico o material por su participación. Los datos de la encuesta serán únicamente utilizados para fines académico-científicos sobre el vegetarianismo en Argentina.

De tener alguna pregunta sobre sus derechos como participante, reclamo o queja relacionada con su participación en este estudio, favor de comunicarse con la Secretaría de Investigación de la Facultad de Ciencias de la Salud de la Universidad Adventista del Plata, al teléfono 0343-4918000, extensión 1236, 1387 o 1383 y correos electrónicos investfcs@uap.edu.ar y/o cienciaytecnica@uap.edu.ar

¡Muchas gracias por su participación!

Al hacer clic en el botón "ACEPTAR", indica que usted tiene 18 años o más y ha leído la información anterior, aceptando voluntariamente a participar del estudio.

*Le, L. T., Sabaté, J., Singh, P. N., & Jaceldo-Siegl, K. (2018). The design, development and evaluation of the vegetarian lifestyle index on dietary patterns among vegetarians and non-vegetarians. Nutrients. https://www.ncbi.nlm.nih.gov/pmc/articles/PMC5986422/

☑ **ACEPTAR**

Siguiente

INDICE DE Calidad de dieta Vegetariana

INSTRUCCIONES

Piense en los **últimos 12 meses de su alimentación** y marque con qué frecuencia consumió los siguientes alimentos.
Le dejamos un **ejemplo de cómo contestar a las preguntas**.

Cuando le preguntemos "¿Cuántas porciones de **granos integrales** consume en un día?" [illegible]

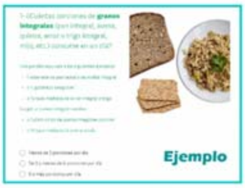

[illegible] "De 3 a menos de 6 porciones por día".

- Menos de 3 porciones por día
- De 3 a menos de 6 porciones por día
- 6 o más porciones por día

A CONTINUACIÓN, COMENZAREMOS CON LA ENCUESTA:

1- ¿Cuántas porciones de **granos integrales** consume en un día?
(pan integral, avena, quinoa, arroz o trigo integral, mijo, etc.)

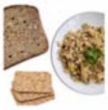

Una porción equivale a los siguientes ejemplos:
- 1 rebanada de pan [illegible] integral
- o 4 galletas integrales
- o ½ taza mediana de [illegible], quinoa o arroz integral cocidos
- o 1 plato chico de pastas integrales cocidas
- o ½ taza mediana de avena

- Menos de 3 porciones por día
- De 3 a menos de 6 porciones por día
- 6 o más porciones por día

2- ¿Cuántas porciones de **legumbres y sus derivados** consume en un día?
(lentejas, porotos, arvejas, habas, chaucha, garbanzo, soja y derivados como leche de soja, tofu, humus, milanesas o hamburguesas de legumbres, etc.)

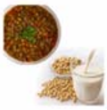

Una porción equivale a los siguientes ejemplos:
- ½ taza mediana de lentejas, porotos, soja, garbanzos o arvejas cocidas
- o 1 milanesa de legumbres grande
- o [illegible] de tofu
- o 4 cucharadas soperas de untable de legumbres (ej. humus)
- o 1 vaso de leche de soja o [illegible] cucharadas soperas de leche de soja en polvo

- Menos de 1 porción por día
- De 1 a menos de 2 porciones por día
- 2 o más porciones por día

3- ¿Cuántas porciones de **verduras** come en un día?
(verduras crudas, verduras cocidas y jugos de verduras 100% naturales)

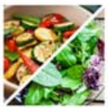

Una porción equivale a los siguientes ejemplos:
- 1 plato grande de verduras de hojas verdes crudas como lechuga, rúcula, achicoria, espinaca, etc.
- o 1 cucharón chico de verduras crudas como tomate, zanahoria, rabanitos, pepino, cebolla, etc.
- o 1 cucharón de verduras cocidas utilizadas en guisos, [illegible], en tartas o empanadas, etc.
- o 1 vaso mediano de jugo de verduras 100% naturales (tomate, zanahoria, [illegible], etc.)

- Menos de 4 porciones por día
- De 4 a menos de 6 porciones por día
- 6 o más porciones por día

4- ¿Cuántas porciones de **frutas** come en un día?

(frutas frescas, deshidratadas, enlatadas, cocidas y jugos de frutas 100% naturales)

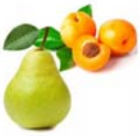

Una porción equivale a los siguientes ejemplos:

- 1 fruta mediana o 2 frutas pequeñas
- o 2 cucharadas soperas de pasas o 1 orejón de pera o durazno
- o 1 vaso mediano de jugos 100% naturales

- Menos de 2 porciones por día
- De 2 a menos de 4 porciones por día
- 4 o más porciones por día

5- ¿Cuántas porciones de **frutos secos y semillas** consume en un día?

(nueces, almendras, castañas, lino, chía, girasol, sésamo, etc.)

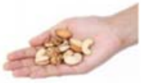

Una porción equivale a los siguientes ejemplos:

- 2 cucharadas soperas de semillas
- o 10 unidades de almendras, nueces o castañas
- o 1 vaso de leche o jugo de frutos secos o semillas

- Menos de 4 porciones por semana
- De 4 porciones por semana a 1 porción por día
- 1 ½ o más porciones por día

6- ¿Cuántas porciones de **aceites vegetales no calentados** (aceite de oliva, girasol, maíz o soja, etc.), **palta y aceitunas** consume en un día?

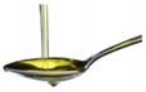

Una porción equivale a los siguientes ejemplos:

- 2 cucharadas chicas (tipo postre) de aceite no calentado o usado en la cocción
- o ½ palta chica
- o 3 cucharadas tipo postre de pasta de aceitunas
- o 20 aceitunas enteras

- Hasta 2 porciones por día
- Más de 2 hasta 4 porciones por día
- Más de 4 porciones por día

7- ¿Cuántas porciones de **lácteos** consume en un día?

(queso, yogur, leche, postres lácteos, etc.)

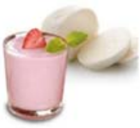

Una porción equivale a los siguientes ejemplos:

- 1 taza grande de leche o yogur
- o 1 rebanada mediana de queso fresco
- o 3 cucharadas soperas de queso untable

- No consumo
- Hasta 2 porciones por día
- Más de 2 porciones por día

8- ¿Cuántas porciones de **huevo** consume en un día?
(hervido, en preparaciones como revuelto, rellenos, tortilla, ensalada, tortas etc.)

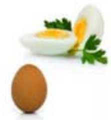

Una porción equivale a los siguientes ejemplos:
- 1 huevo
- o 3 claras

- ◯ No consume
- ◯ Hasta 1 porción por día
- ◯ Más de 1 porción por día

9- ¿Cuántas porciones de **dulces** consume en **una semana**?
(tortas, helados, chocolates, mermeladas, dulces, bebidas azucaradas, etc.)

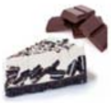

Una porción equivale a los siguientes ejemplos:
- 1 porción de torta
- o 1 cucharada [illegible] de mermelada
- o 2 bochas de helado
- o 4 cuadraditos de chocolate
- o 1 alfajor
- o 1 vaso mediano de gaseosa u otra bebida azucarada

- ◯ Menos de 3 porciones por semana
- ◯ De 3 a 5 porciones por semana
- ◯ Más de 5 porciones por semana

10- ¿Cuántas porciones de **fuentes confiables de vitamina B12** consume en un día?
Incluye: carne, pescado, lácteos, huevos, alimentos fortificados y suplementos

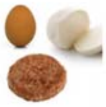

Una porción equivale a los siguientes ejemplos:
- 1 porción chica de carne (vacuna, ave y/o pescado)
- o ½ vaso de leche
- o 2 rebanadas de queso
- o 1 huevo
- o 1 vaso de jugo o leche vegetal comercial fortificada con vitamina B12
- o 1 suplemento de 100 microgramos de vitamina B12 por día
- o 1 suplemento de 1000 microgramos de vitamina B12 por semana que equivale al consumo de 2 o más porciones de suplemento de B12 por día

- ◯ Menos de 1 porción por día
- ◯ 1 porción por día
- ◯ 2 o más porciones por día

11- ¿Cuántas veces en la semana consume **carnes**?
(carne roja, pescado, pollo y carnes procesadas como chorizo, hamburguesa, salchicha, etc.)

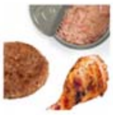

- ◯ No consume
- ◯ Menos de 1 vez por mes hasta 1 vez por semana
- ◯ Más de 1 vez por semana

12- ¿Cuántos **minutos** realiza **de actividad física** en un día? (**si no realiza todos los días** actividad física, **promedie** en un día la actividad semanal que realiza)

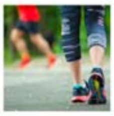

Ejemplos de actividad física (AF) moderada
- Caminata rápida
- Jardinería o tareas domésticas activas
- Trabajos de construcción generales

Ejemplos de actividad física (AF) intensa
- Correr o trotar
- Actividades en el gimnasio
- Desplazamientos rápidos en bicicleta
- Deportes competitivos

- ◯ No realiza actividad física
- ◯ Menos de 30 min por día de AF moderada o menos de 15 min por día de AF intensa
- ◯ 30 min o más por día de AF moderada o 15 min o más por día de AF intensa

13- ¿Cuántos vasos de **agua de 250ml** consume al día?

- ◯ Menos de 4 vasos por día
- ◯ De 4 a 7 vasos por día
- ◯ 8 o más vasos por día

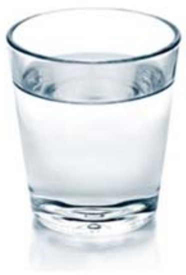

14- ¿Cuántos **minutos** se expone al **sol** (al menos brazos y/o piernas) diariamente **entre las 11 y las 13 hs**.?

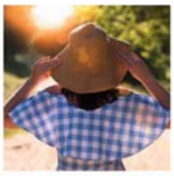

- ◯ Menos de 5 min por día
- ◯ De 5 a menos de 10 min por día
- ◯ 10 min o más por día

15- Marque dónde compra habitualmente los siguientes alimentos. **De cada lugar marque la opción donde más compra**, si cerca o lejos de su casa. Si usted no consume alguno de los alimentos salte a la siguiente opción.

Considere como:

Cerca = menos de 10 cuadras de mi casa **Lejos** = 10 o más cuadras de mi casa

	**Dietetica** Cerca	**Dietetica** Lejos	**Supermercado** Cerca	**Supermercado** Lejos	**Ferias locales** cerca	**Ferias locales** lejos	**Internet**
Leche y/o yogur de vaca fortificados	☐	☐	☐	☐	☐	☐	☐
Leche vegetal fortificada (soja, almendras, coco)	☐	☐	☐	☐	☐	☐	☐
Yogur a base de leches vegetales	☐	☐	☐	☐	☐	☐	☐
Tofu y otros quesos a base de leches vegetales	☐	☐	☐	☐	☐	☐	☐
Hamburguesas de legumbres y/o semillas	☐	☐	☐	☐	☐	☐	☐
Fiambres de origen vegetal	☐	☐	☐	☐	☐	☐	☐
Panificados dulces o postres veganos	☐	☐	☐	☐	☐	☐	☐
Jugos de frutas 100% naturales	☐	☐	☐	☐	☐	☐	☐
Aceite de oliva	☐	☐	☐	☐	☐	☐	☐
Semillas (chía, lino, sésamo, etc.)	☐	☐	☐	☐	☐	☐	☐
Frutos secos (nueces, almendras, etc.)	☐	☐	☐	☐	☐	☐	☐

Anterior | Siguiente

INDICE DE Calidad de dieta Vegetariana

Para los siguientes datos marque una sola opción según corresponda o complete el espacio

1- Edad (años)

2- Especifique:

Peso (kg):

Altura (metros):

3- Sexo:

- Masculino
- Femenino

4- ¿Presenta alguna **enfermedad** diagnosticada por el medico?

- No
- Si, Mencione cuál:

5- ¿Toma alguna **medicación**?

- No
- Si, Mencione cuál:

6- Estado civil

- Soltero/a
- Divorciado/a
- Casado/a
- Viudo/a
- En convivencia

7- Nivel educativo más alto alcanzado

- Primario
- Universitario
- Secundario
- Posgrado
- Terciario

8- Ocupación actual:

9- ¿Dónde vive actualmente? (mencione provincia y ciudad):

10- ¿Qué porcentaje de sus ingresos mensuales utiliza en concepto de alimentación?

Si usted vive con su familia considere los ingresos totales del hogar

0% 100%

11- ¿Cómo clasificaría actualmente su dieta?

Elija la opción que más se adapte o acerque a su dieta

- **Omnívora** (consume carnes rojas, pollo, o pescado)
- **Semi-vegetariano** o **"flexitariano"** (consume carnes rojas, pollo o pescado una vez al mes o más, pero no más de una vez por semana)
- **Pesco vegetariano** (consume pescado u otros organismos acuáticos, pero no consume carnes rojas o pollo)
- **Lacto-ovo vegetariano** (consume huevos y productos lácteos, pero no consume carnes de ningún tipo)
- **Otro** (especificar cuál)
- **Lacto vegetariano** (consume lácteos y derivados, pero no consume huevos, ni carnes)
- **Ovo vegetariano** (consume huevos, pero no consume lácteos, ni carnes)
- **Vegetariano** (no consume carnes, ni lácteos ni huevos)
- **Vegano** (no consume ningún alimento de origen animal y sus derivados como carnes, lácteos, huevos, miel, gelatina, etc.)

12- ¿Hace cuánto que usted mantiene este tipo de dieta?

- Menos de 6 meses
- Más de 12 meses
- Entre 6 y 12 meses

13- ¿Con qué frecuencia fuma?

12- ¿Hace cuánto que usted mantiene este tipo de dieta?

- Menos de 6 meses
- Más de 12 meses
- Entre 6 y 12 meses

13- ¿Con qué frecuencia fuma?

- No fumo
- De 6 a 10 cigarrillos por día
- De 1 a 3 veces por semana
- De 10 a 20 cigarrillos por día
- De 1 a 5 cigarrillos por día
- Más de 20 cigarrillos por día

14- ¿Con qué frecuencia consume alguna bebida alcohólica?

Tenga en cuenta las bebidas alcohólicas que consume junto con las comidas, además de las que consume en ocasiones sociales. Incluya bebidas como whisky, vodka, fernet, vino, cerveza, bebidas espumantes, etc.

- Nunca
- 2 a 3 veces a la semana
- Una o menos veces al mes
- 4 o más veces a la semana
- De 2 a 4 veces al mes

Anterior Siguiente

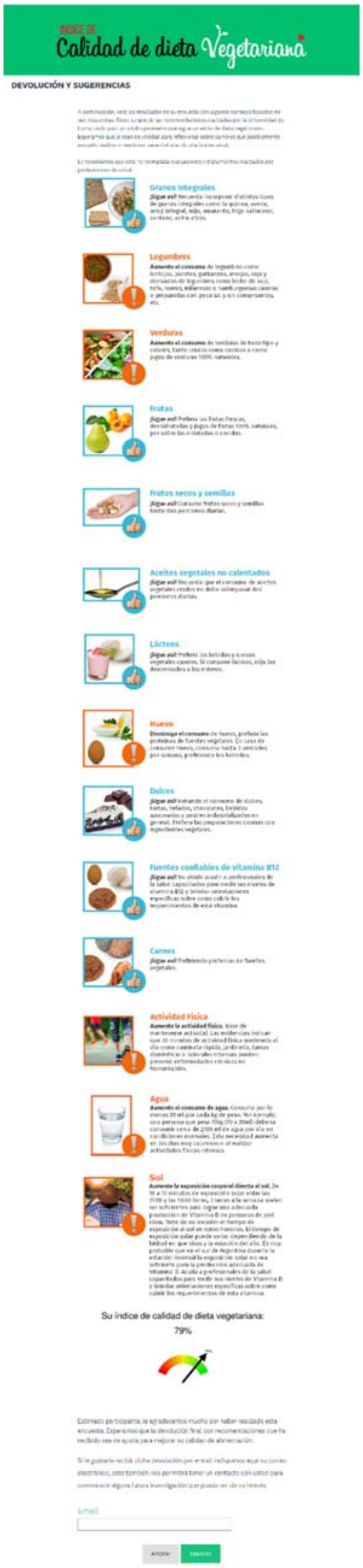
ÍNDICE DE
Calidad de dieta Vegetariana
DEVOLUCIÓN Y SUGERENCIAS
Granos integrales
Legumbres
Verduras
Frutas
Frutos secos y semillas
Aceites vegetales no calentados
Lácteos
Huevo
Dulces
Fuentes confiables de vitamina B12
Carnes
Actividad Física
Agua
Sol
Su índice de calidad de dieta vegetariana:
79%
Email:
